# Antibacterial Activity and Mechanism of Protocatechuic Acid Against Pathogens Isolated from Canine Endometritis

**DOI:** 10.3390/ani16071018

**Published:** 2026-03-26

**Authors:** Xiaoyu Sun, Jingwen Bi, Dongxue Shi, Haiyue Xu, Yuqi Liang, Weitao Dong, Xingxu Zhao, Yong Zhang

**Affiliations:** 1 College of Veterinary Medicine, Gansu Agricultural University, Lanzhou 730070, China; 13515351766@163.com (X.S.); 18894310603@163.com (J.B.); shidx@gsau.edu.cn (D.S.); 1073325010117@st.gsau.edu.cn (H.X.); gloriatalk@outlook.com (Y.L.); dongwt@gsau.edu.cn (W.D.); 2 Gansu Key Laboratory of Animal Generational Physiology and Reproductive Regulation, Lanzhou 730070, China

**Keywords:** protocatechuic acid, canine endometritis, antibacterial mechanism, membrane damage

## Abstract

Canine endometritis is commonly associated with bacterial infections caused by *Escherichia coli* (*E. coli*), *Staphylococcus aureus* (*S. aureus*), and *Streptococcus canis* (*S. canis*), leading to reproductive disorders in dogs. With increasing concern regarding antimicrobial resistance, alternative therapeutic strategies are needed. This study evaluated the in vitro antibacterial activity and underlying mechanisms of protocatechuic acid (PCA) against clinical isolates of these pathogens obtained from dogs diagnosed with endometritis. The antibacterial efficacy of PCA was assessed by determining minimum inhibitory concentrations (MICs), minimum bactericidal concentrations (MBCs), and bacterial growth curves. PCA inhibited the growth of all three pathogens, with MIC values of 4 mg/mL for *E. coli* and *S. aureus* and 2 mg/mL for *S. canis*. The MBCs for *E. coli* and *S. aureus* were equal to their MICs, while the MBC for *S. canis* was twice the MIC, indicating bactericidal activity. Mechanistic analyses demonstrated that PCA disrupted bacterial membrane integrity, induced membrane depolarization, reduced intracellular ATP levels, and increased reactive oxygen species (ROS) accumulation. These effects were supported by SYTO9/PI fluorescence staining and scanning electron microscopy. In conclusion, PCA exhibits notable in vitro antibacterial activity against key pathogens associated with canine endometritis and represents a promising natural antimicrobial candidate.

## Simple Summary

Canine endometritis is a common reproductive disorder in veterinary practice and is frequently associated with bacterial infections. Although antibiotics are widely used for treatment, concerns regarding antimicrobial resistance have prompted the search for alternative therapeutic options. In this study, protocatechuic acid, a natural phenolic compound, was evaluated for its antibacterial activity against *Escherichia coli*, *Staphylococcus aureus*, and *Streptococcus canis* isolated from dogs clinically diagnosed with endometritis. The results demonstrated that PCA effectively inhibited bacterial growth in vitro and exerted bactericidal activity. Mechanistic analyses revealed that PCA disrupted bacterial membrane integrity, altered membrane potential, reduced intracellular ATP levels, and induced reactive oxygen species accumulation. These findings suggest that PCA has potential as a natural antimicrobial agent for the control of canine endometritis and warrant further investigation for localized veterinary applications.

## 1. Introduction

Endometritis refers to inflammatory changes in the endometrial tissue and is one of the most common and challenging disorders affecting the reproductive system of dogs [1]. It often occurs during estrus, mating, abortion, or parturition and is mostly considered the result of ascending infections by pathogenic microorganisms such as *E. coli*, *S. aureus*, and *S. canis*, which impair reproductive success [2]. The incidence of endometritis in infertile bitches has been reported to be relatively high, and the condition can significantly impair normal reproductive function [3]. In severe cases, it can result in persistent reproductive disorders or infertility [4]. Reproductive failure is a major limiting factor for breeding performance and reduces animal welfare. At present, antibiotics are the main treatment methods [5]. However, the indiscriminate use of antibiotics not only promotes the emergence of drug-resistant bacterial strains and reduces the effectiveness of drugs but also brings ecological and public health hazards [6]. Therefore, research on alternative veterinary drugs has become a key issue in veterinary medicine.

Due to their multiple biological activities, natural phenolic acids have attracted increasing attention [7]. PCA is a natural phenolic acid class that exists widely in many kinds of plants and shows multiple pharmacological activities, such as antibacterial and anti-inflammatory activities [8]. There is consensus from multiple in vitro studies that PCA exhibits antibacterial activity. As reported, PCA exhibited remarkable antibacterial activity against *Pseudomonas aeruginosa* [9], *Micrococcus luteus* [10], and *Listeria monocytogenes* [11]. The possible antibacterial mechanism of PCA may involve the disruption of the bacterial metabolic system and the bacterial cell membrane structure. PCA is expected to be a potential natural antimicrobial agent for canine endometritis.

Therefore, the present study aimed to evaluate the in vitro antibacterial activity of PCA against clinically isolated pathogens associated with canine endometritis and to explore its potential membrane-targeting mechanisms. By clarifying its direct antibacterial effects and possible mode of action, this study provides experimental evidence to support further investigation of PCA as a potential antimicrobial agent in veterinary medicine.

## 2. Materials and Methods

### 2.1. Bacteria Used in the Experiment

We conducted clinical examinations on 19 female Beagle dogs with long-term infertility, aged 10–12 months and weighing 8–10 kg, at Qingdao Bolong Experimental Animal Co., Ltd. (Qingdao, China). These dogs had no prior medication history. The diagnosis of endometritis was established based on clinical evaluation combined with ultrasonographic findings, including uterine enlargement or the presence of intrauterine fluid accumulation. In addition, routine hematological and serum biochemical examinations were performed to assess the inflammatory status and general health condition. Following clinical diagnosis, uterine lavage samples were collected under sterile conditions. Specifically, the external genitalia of the female dogs were disinfected with iodine. After disinfection, a double-layer sampling tube was inserted into the vagina of the female dog. When the outer tube reached the cervix, the inner tube was slowly advanced into the uterus. Physiological saline was injected into the uterus to collect uterine flushing fluid, which was subsequently used to isolate and identify bacteria. The purified pathogens (*E. coli*, *S. aureus*, and *S. canis*) were mixed 1:1 with sterile glycerol and stored in sterile cryovials at −80 °C for subsequent use.

### 2.2. Determination of MIC and MBC

The MIC of PCA (Sigma-Aldrich, St. Louis, MO, USA) was determined using the broth dilution method based on the modified protocols of the European Committee for Antimicrobial Susceptibility Testing. The MICs of ampicillin were determined in parallel using the same method and the same representative isolates and served as the positive reference standard for antibacterial activity. Purified *E. coli* and *S. aureus* were inoculated into Mueller–Hinton broth (MHB, Solarbio, Beijing, China), while *S. canis* was cultured in MHB supplemented with 5% sheep serum (Solarbio, Beijing, China) and incubated at 37 °C for 18 h. Bacterial cultures in the logarithmic growth phase were standardized by adjusting the optical density to OD600 = 0.1, corresponding to a final concentration of approximately 1 × 10^6^ CFU/mL, to ensure consistency of the inoculum across all experiments. Antibiotic stock solutions were prepared using MHB (with 5% sheep serum for the *S. canis* group). PCA solutions at varying concentrations were prepared in 96-well plates by adding 50 μL of standardized bacterial suspension and 50 μL of MHB (MHB with 5% sheep serum for *S. canis*). Positive controls were established by adding 50 μL of bacterial suspension and 50 μL of ampicillin solution (serially diluted for MIC determination). Each condition was tested in triplicate. The plates were incubated at 37 °C for 18 h, and optical density (OD) at 600 nm was measured with a microplate reader (Spectramax i3x, Molecular Devices, Shanghai, China). The minimum bactericidal concentration (MBC) of PCA was determined following the MIC assay. After incubation with PCA at the indicated concentrations, aliquots (100 μL) from each treatment group showing no visible bacterial growth were collected and plated onto Brain Heart Infusion (BHI) agar plates. The plates were incubated at 37 °C for 24 h, and bacterial growth was assessed by colony formation. The MBC was defined as the lowest concentration of PCA at which no bacterial colonies were observed on the agar plates.

### 2.3. Bacterial Growth Curve

Three bacteria were separately inoculated into PCA at different concentrations (0, 1/4 MIC, 1/2 MIC, MIC, and 2 MIC) using bacterial suspensions adjusted to approximately 1 × 10^6^ CFU/mL. Cultures were shaken at 37 °C at 180 rpm. The OD_600_ of the bacterial cultures was analyzed every 2 h. For each measurement, a 200 μL aliquot was transferred to a 96-well plate, with readings taken on a microplate reader (Spectramax i3x, Molecular Devices, Shanghai, China). This process was continued for 24 h, and growth curves were plotted accordingly.

### 2.4. Determination of Membrane Potential

Bacterial cells (*E. coli*, *S. aureus*, and *S. canis*) were harvested by centrifugation (4 °C, 5000× *g*, 5 min), washed three times with phosphate-buffered saline (PBS), and adjusted to an OD600 of 0.1 in PBS. With sterile PBS as the solvent, PCA was prepared and added to the bacterial suspensions at final concentrations of MIC and 2MIC, respectively. Bacterial suspensions containing sterile PBS without PCA were used as the control. After incubation at 37 °C for 2 h, a 0.5 μM 3,3-Dipropylthiadicarbocyanine iodide [DiSC_3_(5), Invitrogen, Carlsbad, CA, USA] fluorescent probe was added and incubated for 30 min. Subsequently, 200 μL of each bacterial suspension was transferred into black-walled 96-well plates. Each sample was tested in triplicate, and fluorescence intensity was measured using a multifunctional microplate reader (Clariostar, BMC Labtech, Ortenberg, Germany) at excitation 622 nm and emission 670 nm. With PBS as the solvent, PCA was prepared and added to the bacterial suspensions at final concentrations of MIC and 2MIC, respectively. Bacterial suspensions containing PBS without PCA were used as the control.

### 2.5. Determination of ATP Levels

Bacterial suspensions (approximately 1 × 10^6^ CFU/mL) were prepared as described in Section 2.4 and incubated with PCA at final concentrations of 0 (PBS-treated control), MIC, and 2MIC for 2 h. The bacterial suspensions were then collected by centrifugation (4000× *g*, 5 min, 4 °C), washed three times with PBS, and processed according to the instructions of the ATP assay kit (Solarbio, Beijing, China). Extraction buffer was added, and cells were disrupted by sonication (ice bath, 200 W power, 2 s pulses with 1 s intervals for a total of 1 min). The lysates were centrifuged (10,000× *g*, 4 °C, 10 min), and the supernatants were transferred to fresh tubes. After thorough mixing with 500 μL of chloroform and subsequent centrifugation, the supernatants were collected for analysis. The UV spectrophotometer (UV6000, Metash, Shanghai, China) was preheated for 30 min at 340 nm. Reagents were added to 1 mL quartz cuvettes, mixed, and absorbance was recorded at 10 s (A1 measurement and standard). After incubation at 37 °C for 3 min, absorbance values were recorded again (A2, sample and standard). ATP content was calculated using the following formula: ATP content (μmol/g) = ΔA_sample ÷ (ΔA_standard ÷ C_standard) × V_extraction ÷ W.

### 2.6. Determination of ROS Level

The level of intracellular ROS in *E. coli*, *S. aureus*, and *S. canis* was determined using a modified method described by Cutro [12]. Following preparation as described in Section 2.4, bacterial suspensions (approximately 1 × 10^6^ CFU/mL) were exposed to PCA at final concentrations of 0 (PBS-treated control), MIC, and 2MIC in sterile PBS for 30 min at 37 °C. The probe 2′,7′-Dichlorodihydrofluorescein Diacetate (DCFH-DA, Sigma-Aldrich, St. Louis, MO, USA) was added to the bacterial suspension, gently mixed, and then incubated in the dark at 37 °C for 30 min. After centrifugation (4 °C, 500× *g*, 5 min), the pellets were resuspended and washed three times with PBS, followed by incubation in the dark. A drop of the bacterial suspension was placed on a microscope slide and observed under a fluorescence microscope (IX73, OLYMPUS, Tokyo, Japan) equipped with FITC filters (excitation 488 nm, emission 525 nm). Images were captured for documentation. Bacteria exhibiting green fluorescence were considered ROS positive, and fluorescence intensity was quantified using ImageJ 1.43 (NIH, Bethesda, MD, USA).

### 2.7. Bacterial Cell Membrane Integrity

Following the protocol described by Guo [13], this study evaluated the effect of PCA on the membrane integrity of *E. coli*, *S. aureus*, and *S. canis* using the SYTO9/PI double-staining technique. Bacterial suspensions (approximately 1 × 10^6^ CFU/mL) were prepared as described in Section 2.4. With sterile PBS as the solvent, PCA was prepared and added to the bacterial suspensions at final concentrations of MIC and 2MIC, respectively, while bacterial suspensions containing sterile PBS without PCA served as the control. SYTO9 and PI solutions were premixed at a 1:1 (*v*/*v*) ratio. After incubation with PCA at 37 °C for 2 h, a 3 μM SYTO9/PI probe (Fushebio, Shanghai, China) was added and incubated in the dark for 10 min. Bacteria were washed twice, pelleted by centrifugation (4 °C, 5000× *g*, 5 min), and resuspended in saline. Drops of suspension (2 μL) were placed on slides and visualized under confocal laser scanning microscopy (CLSM, LSM900; Carl Zeiss, Jena, Germany), and images were recorded.

### 2.8. Scanning Electron Microscopy (SEM)

The morphological alterations of three bacteria following PCA treatment were examined by SEM according to the method described by Ryu [14]. Grouping and bacterial suspension preparation followed the procedures described in Section 2.4. Bacterial suspensions (approximately 1 × 10^6^ CFU/mL) were incubated with PCA at final concentrations of 0 (PBS-treated control), MIC, and 2MIC in sterile PBS at 37 °C for 4 h. Cells were then harvested by centrifugation (3000× *g*, 5 min) at room temperature. Secondary fixation was performed overnight at 4 °C with 2.5% glutaraldehyde, followed by three washes with PBS and dehydration through a graded ethanol series (50%, 80%, 90%; 20 min each). Pre-drying was completed with 100% ethanol. Samples were then dried with CO_2_ in a critical point dryer for 20 min, mounted on SEM stubs, sputter-coated with gold–palladium, and observed under high vacuum at 10 kV using a scanning electron microscope (Quattro, FEI, Hillsboro, OR, USA). Representative images were captured for documentation.

### 2.9. Data Statistics and Analysis

All experiments were performed using three independent biological replicates. For each biological replicate, measurements were performed in technical triplicate where applicable. Data are presented as mean ± SEM. Statistical analyses were performed using SPSS Statistics 26 (IBM, Armonk, NY, USA). Prior to ANOVA, the normality of the residuals was assessed using the Shapiro–Wilk test, and the homogeneity of variance was evaluated using Levene’s test. The results of these tests for all variables are provided in Supplementary Table S1. All variables met the assumptions of normality and homogeneity of variance (*p* > 0.05). Differences among groups were analyzed by one-way ANOVA, and *p* < 0.05 was considered statistically significant. Statistical charts were generated using OriginPro 2021 (OriginLab, Northampton, MA, USA).

## 3. Results

### 3.1. Antibacterial Activity of PCA

**Figure 1:**
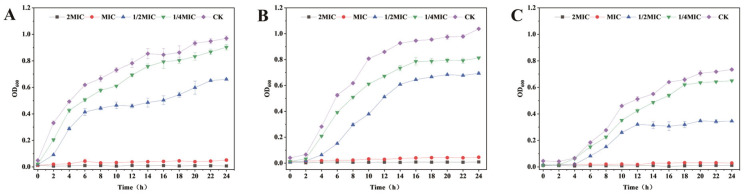
Growth curve analysis of *E. coli* (**A**), *S. aureus* (**B**), and *S. canis* (**C**) with various concentrations of PCA. Bar graphs show the mean ± SEM (*n* = 3).

As shown in Table 1, the MICs of PCA against *E. coli*, *S. aureus*, and *S. canis* were 4, 4, and 2 mg/mL, respectively, whereas the MICs of ampicillin against all three species were 0.0625 mg/mL. In addition, the MBC values of PCA against *E. coli*, *S. aureus*, and *S. canis* were all 4 mg/mL. For ampicillin, the MBC values against *E. coli*, *S. aureus*, and *S. canis* were 0.125, 0.125, and 0.0625 mg/mL, respectively. The effects of PCA with different concentrations on the growth of three bacteria in 24 h were further studied by growth curve assay. The data provide evidence that the control group of the above three kinds of bacteria had a typical growth curve in 24 h. Meanwhile, complete growth inhibition of *E. coli*, *S. aureus*, and *S. canis* was observed at the MIC and 2MIC of PCA. In addition, 1/2 MIC of the test samples reduced the bacterial growth almost completely. In the case of 1/4 MIC, the growth of these bacteria was always slower than the respective untreated control (Figure 1). Therefore, the experimental results indicate that PCA can effectively inhibit the proliferation of three bacteria and exert antibacterial effects.

### 3.2. Effects of PCA on Membrane Potential

**Figure 2:**
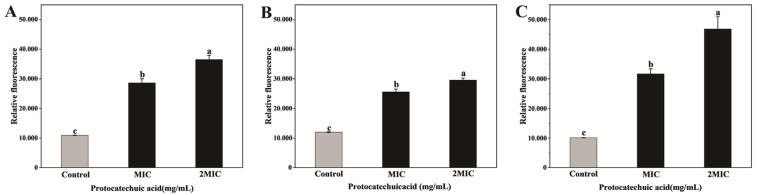
Effects of PCA on the membrane potential of *E. coli* (**A**), *S. aureus* (**B**), and *S. canis* (**C**). Bar graphs show the mean ± SEM (*n* = 3). Different letters indicate statistically significant differences (*p* < 0.05).

As shown in Figure 2, treatment with PCA resulted in significantly elevated membrane potential fluorescence values in three bacteria compared to the control group (*p* < 0.05). This finding indicates that the bacterial cell membranes underwent depolarization, which contributed to the inhibition of bacterial growth.

### 3.3. Effects of PCA on ATP Level

**Figure 3:**
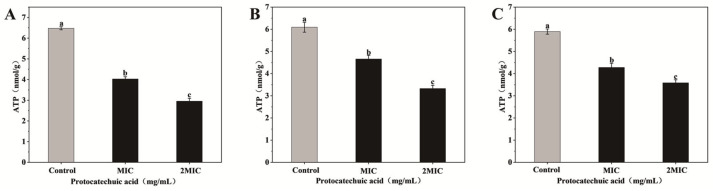
Effects of PCA on intracellular ATP of *E. coli* (**A**), *S. aureus* (**B**), and *S. canis* (**C**). Bar graphs show the mean ± SEM (*n* = 3). Different letters indicate statistically significant differences (*p* < 0.05).

As illustrated in Figure 3, exposure to PCA led to a notable decrease in intracellular ATP levels in three bacteria. For *E. coli*, the baseline ATP concentration was 6.48 μmol/g; upon treatment with PCA at MIC and 2MIC, concentrations decreased to 4.03 μmol/g and 2.95 μmol/g, respectively. For *S. aureus*, ATP content declined from 6.09 μmol/g to 4.66 μmol/g and 3.33 μmol/g at MIC and 2MIC, respectively. Likewise, in *S. canis*, ATP levels dropped from 5.89 μmol/g to 4.28 μmol/g and 3.58 μmol/g under the same conditions. Compared with the control group, the ATP levels in the experimental group were significantly reduced (*p* < 0.05).

### 3.4. Effects of PCA on ROS Levels

**Figure 4:**
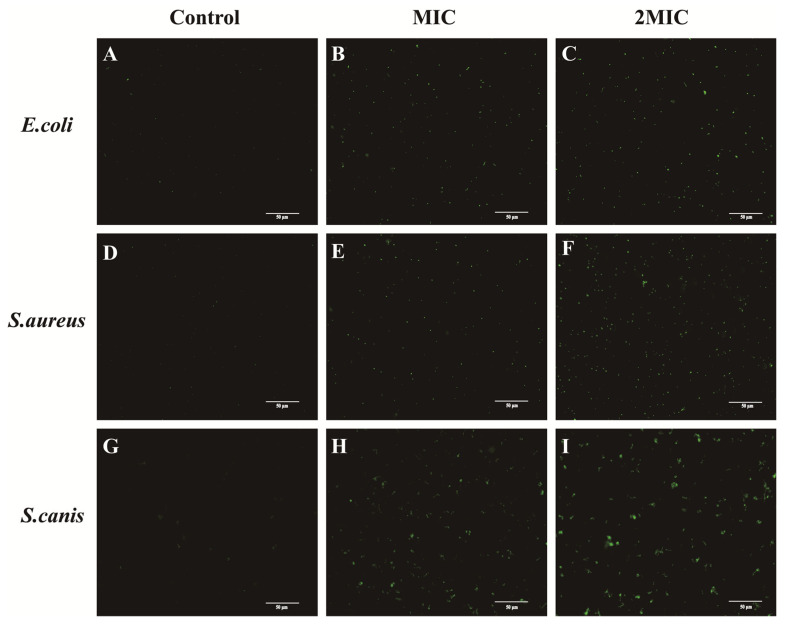
Fluorescent images of ROS in *E. coli*, *S. aureus*, and *S. canis* of different treatment groups. (**A**) *E. coli* control group; (**B**) *E. coli* treated with MIC; (**C**) *E. coli* treated with 2MIC; (**D**) *S. aureus* control group; (**E**) *S. aureus* treated with MIC; (**F**) *S. aureus* treated with 2MIC; (**G**) *S. canis* control group; (**H**) *S. canis* treated with MIC; (**I**) *S. canis* treated with 2MIC.

**Figure 5:**
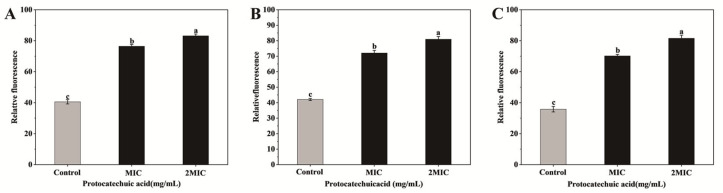
Effects of PCA on ROS levels in *E. coli* (**A**), *S. aureus* (**B**), and *S. canis* (**C**). Bar graphs show the mean ± SEM (*n* = 3). Different letters indicate statistically significant differences (*p* < 0.05).

As presented in Figure 4 and Figure 5, the ROS fluorescence intensity increased following PCA treatment at both MIC and 2MIC for all three pathogens. Specifically, for *E. coli*, baseline fluorescence was 40.58, rising to 76.42 and 83.12 at MIC and 2MIC, respectively. For *S. aureus*, fluorescence increased from 42.08 (control) to 72.06 (MIC) and 80.90 (2MIC). *S. canis* displayed an increase from 35.81 (control) to 70.21 (MIC) and 81.57 (2MIC). This trend demonstrates that PCA induces a concentration-dependent rise in intracellular ROS levels, with statistically significant increases compared to controls (*p* < 0.05).

### 3.5. Effects of PCA on Cell Membrane Damage

**Figure 6:**
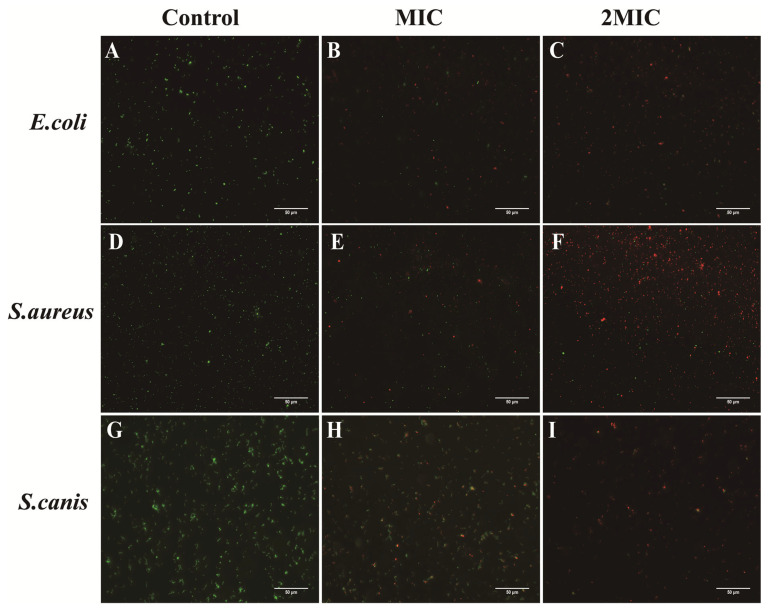
Effects of PCA on the membrane integrity of *E. coli*, *S. aureus*, and *S. canis* under different treatment groups. (**A**) *E. coli* control group; (**B**) *E. coli* treated with MIC; (**C**) *E. coli* treated with 2MIC; (**D**) *S. aureus* control group; (**E**) *S. aureus* treated with MIC; (**F**) *S. aureus* treated with 2MIC; (**G**) *S. canis* control group; (**H**) *S. canis* treated with MIC; (**I**) *S. canis* treated with 2MIC. Green fluorescence indicates live cells, while red fluorescence indicates membrane-damaged cells.

**Figure 7:**
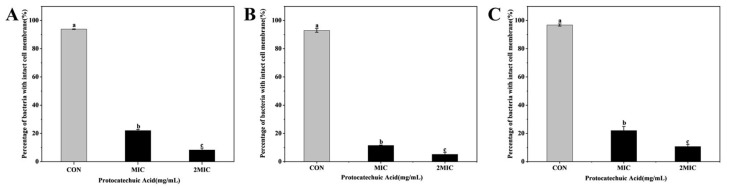
Effects of PCA on the percentage of *E. coli* (**A**), *S. aureus* (**B**), and *S. canis* (**C**) with intact cell membranes. Bar graphs show the mean ± SEM (*n* = 3). Different letters indicate statistically significant differences (*p* < 0.05).

As indicated in Figure 6, all control groups of *E. coli*, *S. aureus*, and *S. canis* (Figure 6A,D,G) displayed only green fluorescence, consistent with intact bacterial cell membranes. After treatment with PCA at MIC, some cells exhibited localized red fluorescence, suggesting partial compromise of membrane integrity (Figure 6B,E,H). At 2MIC of PCA, green fluorescence was almost completely absent, while red fluorescence was markedly increased (Figure 6C,F,I), indicative of extensive bacterial cell membrane damage in a concentration-dependent manner. Quantitative analysis of the PI/SYTO9 fluorescence staining is shown in Figure 7. In the control groups, the percentage of membrane-intact bacteria remained high, indicating preserved cell membrane integrity. Following treatment with PCA at MIC, the proportion of membrane-intact cells was significantly reduced compared with the control group (*p* < 0.05). Upon exposure to PCA at 2MIC, the percentage of membrane-intact bacteria further decreased and reached the lowest level among all groups (*p* < 0.05). These results demonstrate that PCA disrupts bacterial membrane integrity in a clear concentration-dependent manner in *E. coli*, *S. aureus*, and *S. canis*.

### 3.6. SEM Observations

**Figure 8:**
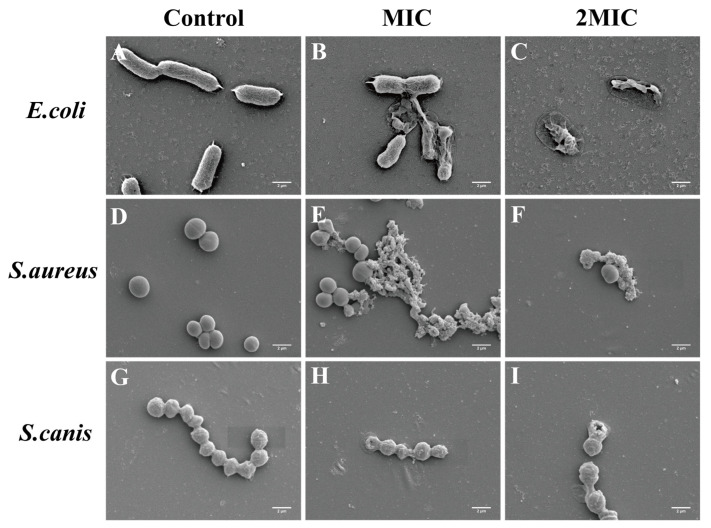
Effects of PCA on the cell morphology of *E. coli*, *S. aureus*, and *S. canis* using SEM. (**A**) *E. coli* control group; (**B**) *E. coli* treated with MIC; (**C**) *E. coli* treated with 2MIC; (**D**) *S. aureus* control group; (**E**) *S. aureus* treated with MIC; (**F**) *S. aureus* treated with 2MIC; (**G**) *S. canis* control group; (**H**) *S. canis* treated with MIC; (**I**) *S. canis* treated with 2MIC.

SEM analysis was used to assess morphological alterations in *E. coli*, *S. aureus*, and *S. canis* after PCA exposure (Figure 8). Untreated *E. coli* displayed a regular rod-shaped structure. Upon exposure to PCA at MIC and 2MIC, pronounced disruption of bacterial cell membrane integrity was observed, including mild contraction, leakage of intracellular contents, and aggregation (Figure 8B,C). Untreated *S. aureus* maintained a smooth, spherical morphology; after PCA treatment, severe membrane and morphological damage ensued, leading to cell surface wrinkling, content leakage, and irregularity (Figure 8E,F). For *S. canis*, the untreated cells exhibited characteristic chain and plump oval forms, but PCA exposure at MIC and 2MIC led to wrinkled cell surfaces, disrupted membranes, and evident leakage of intracellular contents (Figure 8H,I).

## 4. Discussion

In the present study, PCA exhibited notable in vitro antibacterial activity against *E. coli*, *S. aureus*, and *S. canis* isolated from the uterine contents of dogs clinically diagnosed with endometritis. Although the MIC values of PCA were higher than those of ampicillin, PCA consistently exhibited bactericidal activity under standardized in vitro conditions, supporting its potential as a plant-derived antimicrobial candidate. The minimum bactericidal concentrations (MBCs) for *E. coli* and *S. aureus* were identical to their respective minimum inhibitory concentrations (MICs), while the MBC for *S. canis* was twice the MIC, confirming that PCA exerts bactericidal rather than merely bacteriostatic effects. Previous studies have reported antibacterial activities of several natural compounds against these pathogens. For example, Rawangkan [15] reported an MIC of 25 mg/mL for caffeic acid against *E. coli*, while the MIC for curcumin against *S. aureus* ranged from 5 to 10 mg/mL [16]. Additionally, Ebani [17] reported that sage extract has antibacterial activity against *S. canis*, with an MIC value of 16.26 mg/mL. In the present study, PCA demonstrated considerable in vitro antibacterial activity. Although direct comparisons across studies are limited by variations in bacterial strains and experimental methods, within the context of plant-derived compounds reported at mg/mL concentrations, PCA exhibited antibacterial activity within a similar range. This positions PCA as a promising candidate among natural antimicrobial agents. Its potent efficacy against the key pathogens associated with endometritis underscores its potential for further development as a strategic option for controlling this condition.

To elucidate the antibacterial mechanism of PCA against the three bacterial strains, several key physiological parameters were examined, including membrane potential, intracellular ATP and ROS concentrations, cell membrane integrity, and cellular morphology.

Alterations in bacterial cell membrane potential, including depolarization and hyperpolarization, have been widely recognized as important indicators of membrane damage [18]. In the present study, PCA treatment induced significant depolarization of the bacterial cell membranes in *E. coli*, *S. aureus*, and *S. canis*, suggesting that PCA disrupts membrane barrier function. Membrane depolarization caused by natural compounds has been identified as a major mechanism underlying antibacterial activity. Consistent with our findings, Rowaiye [19] reported that extracts of basil, white sage, and sweet locust induced bacterial membrane depolarization, while Wu [20] demonstrated that PCA caused membrane depolarization in *Yersinia enterocolitis*, ultimately leading to bacterial death. Collectively, these results indicate that PCA-mediated membrane depolarization may impair essential cellular functions and contribute to bacterial growth inhibition and death.

ATP plays a central role in cellular energy metabolism, macromolecule synthesis, and enzyme activity, and depletion of intracellular ATP is a well-established marker of cellular damage [21]. In this study, PCA treatment significantly reduced intracellular ATP levels in all three bacterial species. This observation is consistent with previous reports, such as the ATP depletion observed in *Pseudomonas aeruginosa* (*P. rettgeri*) following exposure to sanguinarine [22]. Based on earlier studies, the PCA-induced reduction in ATP content may be attributed to two primary mechanisms: inhibition of bacterial respiration leading to disrupted ATP synthesis [23] and increased membrane permeability resulting in ATP leakage due to membrane damage [24]. These findings further support the notion that PCA interferes with bacterial energy metabolism through membrane-associated mechanisms.

ROS generation represents another important pathway of antibacterial action [25]. Using DCFH-DA fluorescence as an indicator, this study demonstrated that PCA significantly increased intracellular ROS levels in *E. coli*, *S. aureus*, and *S. canis*. Similar effects have been reported for other natural compounds; for example, thymol has been shown to elevate ROS levels in *P. rettgeri*, disrupt membrane permeability, and cause intracellular damage [26]. These results were generally consistent with the results of other analyses [27]. These findings suggest that a key component of PCA’s antibacterial activity lies in its capacity to trigger intracellular ROS accumulation.

To further assess membrane integrity, SYTO9/PI double staining was employed. In control cells, intact membranes allowed SYTO9 binding and green fluorescence, whereas PCA-treated cells exhibited a marked shift toward red PI fluorescence, indicating compromised membrane integrity [28]. At MIC concentrations, PCA caused a significant reduction in green fluorescence accompanied by increased red fluorescence, while treatment at 2MIC resulted in predominant PI staining, reflecting extensive membrane damage. These dose-dependent effects are consistent with previous studies reporting membrane disruption induced by natural antimicrobial compounds. SEM observations further supported the fluorescence staining results. Bacterial cells exposed to PCA at MIC displayed noticeable surface roughening, whereas treatment at 2MIC caused pronounced membrane rupture and substantial leakage of intracellular contents. Similar dose-dependent membrane damage has been reported in *Cryptococcus* following sanguinarine exposure [29] and in Methicillin-resistant *Staphylococcus aureus* (MRSA) treated with oregano essential oil [30], both of which resulted in severe membrane disruption and leakage of cellular materials. Combined SYTO9/PI staining and SEM analyses show that PCA damages bacterial membrane integrity and permeability in a concentration-dependent manner. This progressive membrane damage is a key step in PCA’s antibacterial action and leads to loss of cellular homeostasis and bacterial death.

Compared with many other plant-derived antimicrobial agents, the membrane-targeting activity of PCA may represent characteristics that warrant further exploration. PCA is a small phenolic acid with a well-defined chemical structure. Its structural simplicity may contribute to experimental reproducibility, and it has been reported to induce concentration-dependent membrane damage, which facilitates mechanistic analysis [31]. In contrast to complex plant extracts with heterogeneous and variable compositions, using a single, chemically characterized compound has been reported to allow clearer associations between antibacterial effects and specific cellular targets [32]. Importantly, previous studies have applied multiple membrane-related functional assays in parallel to establish mechanistic frameworks linking concentration-dependent membrane damage to bactericidal activity. It has been suggested that, because membrane disruption represents a broad antibacterial mechanism that is generally less species-specific, compounds like PCA may possess mechanistic characteristics that are less prone to rapid resistance development [33]. Taken together, these properties suggest that PCA offers a stable, reproducible antibacterial effect with a lower likelihood of resistance, highlighting its potential as a promising candidate for future therapeutic applications.

In this study, PCA treatment caused consistent, concentration-dependent increases in intracellular ROS levels, along with reductions in ATP content, membrane potential, and membrane integrity. The convergence of these independent indicators suggests that oxidative stress, membrane disruption, and impairment of cellular energy metabolism collectively contribute to its antibacterial activity. The bactericidal effect was further confirmed by MBC determination, which is consistent with the irreversible membrane damage and metabolic disturbance observed in fluorescence staining and ultrastructural analyses. Together, these findings indicate that PCA primarily targets the bacterial membrane and associated energy metabolism at the cellular level. The relatively high MIC values observed in this study suggest that PCA may be more suitable for localized rather than systemic application [34]. Local delivery strategies, such as vaginal or intrauterine administration, could achieve effective concentrations at the infection site while minimizing systemic exposure, consistent with current veterinary approaches for managing uterine infections [35]. Collectively, these findings not only demonstrate reproducible antibacterial activity against key pathogens associated with canine endometritis but also advance mechanistic insight into membrane-targeting antibacterial strategies within this specific clinical context.

Building on this foundational work, future studies should explore the specific molecular targets of PCA within the canine reproductive tract microenvironment. Considering the dynamic reproductive tract microbiota across different physiological stages may help clarify how PCA interacts with both pathogenic and commensal microorganisms. This approach would provide a clearer mechanistic understanding and support the development of optimized formulations and targeted delivery strategies for localized infections such as canine endometritis.

## 5. Conclusions

In this study, PCA exhibited potent in vitro antibacterial activity against clinical isolates of *E. coli*, *S. aureus*, and *S. canis* obtained from the uterine content of dogs with endometritis. The findings support the notion that PCA exerts bactericidal effects primarily through disruption of bacterial membrane function, providing a mechanistic basis for its antimicrobial action. While these results indicate that PCA is a potential candidate for the management of canine endometritis, further investigations are required to evaluate its clinical applicability, safety, and therapeutic efficacy in vivo.

## Figures and Tables

**Table 1:** MICs and MBCs of PCA and ampicillin against different bacterial strains.

	Protocatechuic Acid (mg/mL)		Ampicillin (mg/mL)	
	MIC	MBC	MIC	MBC
*S. canis*	2	4	0.0625	0.0625
*E. coli*	4	4	0.0625	0.125
*S. aureus*	4	4	0.0625	0.125

## Data Availability

The data presented in this study are contained within the article.

## Supplementary Materials

The following supporting information can be downloaded at: https://www.mdpi.com/article/10.3390/ani16071018/s1.

## Abbreviations

The following abbreviations are used in this manuscript:

PCA	Protocatechuic acid
*E. coli*	*Escherichia coli*
*S. aureus*	*Staphylococcus aureus*
*S. canis*	*Streptococcus canis*
ROS	reactive oxygen species
MIC	Minimum inhibitory concentration
MBC	Minimum bactericidal concentration
PBS	Phosphate-buffered saline
CLSM	Confocal laser scanning microscopy
SEM	Scanning electron microscopy
MHB	Mueller–Hinton broth
CFU	Colony-forming unit
OD	Optical density
DiSC_3_(5)	3,3-Dipropylthiadicarbocyanine iodide
DCFH-DA	2′,7′-Dichlorodihydrofluorescein diacetate
